# Personal Liberty

**Published:** 1909-04

**Authors:** G. B. Dorrell

**Affiliations:** Ex-President Southwest Missouri Medical Society, Republic, Mo.


For Texas Medical Journal.

### Personal Liberty.

BY G. B. DORRELL, M. D.,
Ex-President Southwest Missouri Medical Society, Republic, Mo.

Above all else the founders of this Republic dreaded, as the most dangerous foe to freedom, the government. So strong was this dread, so dearly prized was personal liberty, that not even the authority of Washington and the lofty intellect of Hamilton could render the centralizing Federal party popular. Advocating personal liberty, Jefferson came into office with practically the entire people at his back; and for many years the principles he inculcated held his party in power.

The centrifugal impulse culminated in the secession of States, and since that time the centripetal forces have been dominant. During the last half century we have made enormous strides toward paternalism in our government, and Thomas Jefferson would be, indeed, appalled could he rise and take cognizance of our present state. Each and every step in this direction has been at the expense of the personal liberties of the citizens. However praiseworthy many of the newer laws may be in some respects, it can not be denied that the curtailment of personal privilege has too frequently progressed to a preposterous point. We need go no further than the laws by which medical practice is regulated. Those of California practically exclude all practitioners excepting recent graduates—and quacks. Another Western State expressly forbids all graduates prior to 1893 appearing for examination! The unfortunate gentlemen are not even allowed an opportunity to show their fitness for being entrusted with the lives of sick citizens.

In another direction the abridgment of privilege seems to have approached the absurd. By resolution of the Kentucky State Medical Society, the official organ of that association is forbidden to publish the advertisements of any firm any one of whose products has not as yet received the approval of the Council of Chemistry and Pharmacy. Even products that have been approved by the Council are thus excluded if their manufacturers list anything else for sale. This is assuredly the limit—almost.

We heartily approve of the resolution; all the more since it forbids the advertising of all medical supplies in the Kentucky State Medical Journal. Not one of the firms whose products have been approved by the Council confines its work to the products thus approved, so that manufacturing chemistry is excluded *in toto*. This is a long step toward the one only proper position for an official organ—the exclusion of all advertising. Meanwhile any medical journal that is really independent will continue to advertise *all honest goods* offered by honest manufacturers.

One curious thing seems to have been overlooked by those who have been blaming Editor-Secretary Simmons, as the grand high Pooh-bah of the machine now dominating the A. M. A. Why overlook Billings? Frank Billings has the reputation of having attended more multimillionaire trust magnates to their graves than any other doctor in America. It seems evident that Frank has acquired more than fees from this debasing association, and that the itch for domination, as well as familiarity with trust methods, have been imparted to the doctor. His apparent willingness to have the ex-homeo take an allopathic share *of the hard knocks* favors this surmise.

Twenty-one years ago the great body of the medical profession, then truly represented by the A. M. A., rose in revolt against Eastern arrogance, and asserted its sturdy independence, its equality in privilege and in qualifications with the dwellers along the Atlantic, who were then unaware that America had extended beyond the Alleghenies. But the leaders of that famous revolt are gone. Davis is dead; Cole is dead; Shoemaker has grown old; and a new Moses is about due to arrive. Who will it be, who shall voice the indignation of the free and independent medical profession, and arouse the revolt that will sweep these misrepresentatives out of office?

American Medical Association.—Members of the Medical Society of the Missouri Valley and the Medical Association of the Southwest will be interested in plans that are maturing for a joint excursion train to Atlantic City in June, to attend the annual meeting of the American Medical Association. The special train will start from St. Louis via the Big Four and C. & O. railway, stopping over at Hot Springs, Va., where the delegates will be entertained at luncheon at the famous "Homestead" hotel. Special rates will be in effect, including a boat trip to New York City, and returning either via Niagara Falls or Washington, D. C. By combining the forces of the two societies a special train will be secured, on which no one but doctors and their families will be permitted to ride, thus affording the maximum degree of pleasure and comfort. Members of other medical societies are cordially invited to join the party. Address Dr. Chas. W. Fassett, St Joseph, Mo.

---

Dr. F. B. Hogg of Houston died March 21 (ult.), after a brief illness. He was a well known and popular physician.

---

Dr. W. H. Minton has removed from Ledbetter to Missouri City, Texas.

---

Register of the State Board of Medical Examiners.—The Secretary, Dr. M. E. Daniel, Honey Grove, Texas, has filed with the Secretary of State at Austin a copy of the official record of registered physicians of the State—"nearly 7000." There are three classes of licentiates: those whose credentials have been verified, those who have been examined, and those holding license from other States, licensed by the Texas Board by "reciprocity."

Dr. Daniel will confer an additional benefaction if he will classify the "schools," as there seems to be a wide divergence of opinion as to the number of each. The *Texas State Journal of Medicine* puts the "regulars" at 3322, members of the State Medical Association, and "over 3600" nonmembers, making over 6900 regulars, and says that there are "approximately 175 Eclectics, 105 Homeopaths, 30 Physio-Medicals and 70 Osteopaths" (January number, page 216), while Dr. Braswell of the State Board said, before the Senate committee, that he represented over one thousand of his "school." He is a Physio-Med., I believe.

